# Structural Studies of Epithelial Mesenchymal Transition Breast Tissues

**DOI:** 10.1038/s41598-020-58932-5

**Published:** 2020-02-06

**Authors:** S. N. Mohd Sobri, S. F. Abdul Sani, Siti Norbaini Sabtu, L. M. Looi, S. F. Chiew, Dharini Pathmanathan, Sirinart Chio-Srichan, D. A. Bradley

**Affiliations:** 1 Department of Physics, Faculty of Science, University of Malaya, Kuala Lumpur, 50603 Malaysia; 2 Department of Pathology, Faculty of Medicine, University of Malaya, Kuala Lumpur, 50603 Malaysia; 3 Institute of Mathematical Sciences, Faculty of Science, University of Malaya, Kuala Lumpur, 50603 Malaysia; 4 Synchrotron Light Research Institute (Public Organization), Nakhon Ratchasima, 30000 Thailand; 5 Centre for Biomedical Physics, Sunway University, Petaling Jaya, Kuala Lumpur, 46150 Malaysia; 6 Department of Physics, University of Surrey, Guildford, GU2 7XH UK

**Keywords:** Molecular biophysics, Physics

## Abstract

At the supramolecular level, the proliferation of invasive ductal carcinoma through breast tissue is beyond the range of standard histopathology identification. Using synchrotron small angle x-ray scattering (SAXS) techniques, determining nanometer scale structural changes in breast tissue has been demonstrated to allow discrimination between different tissue types. From a total of 22 patients undergoing symptomatic investigations, different category breast tissue samples were obtained in use of surgically removed tissue, including non-lesional, benign and malignant tumour. Structural components of the tissues were examined at momentum transfer values between q = 0.2 nm^−1^ and 1.5 nm^−1^. From the SAXS patterns, axial d-spacing and diffuse scattering intensity were observed to provide the greatest discrimination between the various tissue types, specifically in regard to the epithelial mesenchymal transition (EMT) structural component in malignant tissue. In non-lesional tissue the axial period of collagen is within the range 63.6–63.7 nm (formalin fixed paraffin embedded (FFPE) dewaxed) and 63.4 (formalin fixed), being 0.9 nm smaller than in EMT cancer-invaded regions. The overall intensity of scattering from cancerous regions is a degree of magnitude greater in cancer-invaded regions. Present work has found that the d-spacing of the EMT positive breast cancer tissue (FFPE (dewaxed)) is within the range 64.5–64.7 nm corresponding to the 9^th^ and 10^th^ order peaks. Of particular note in regard to formalin fixation of samples is that no alteration is observed to occur in the relative differences in collagen d-spacing between non-lesional and malignant tissues. This is a matter of great importance given that preserved-sample and also retrospective study of samples is greatly facilitated by formalin fixation. Present results indicate that as aids in tissue diagnosis SAXS is capable of distinguishing areas of invasion by disease as well as delivering further information at the supramolecular level.

## Introduction

In spite of the enormous forward leaps made in treatment regimens, breast cancer remains a leading cause of death among women worldwide, typically attributable to late detection of the disease^1^. In the absence of major research breakthroughs, the probability for the foreseeable future is that female breast cancer cases are going to escalate^2^. Metastatic disease is accountable for the majority of cancer-induced mortality.

Diagnosis of breast tumour can result from breast screening (eg in asymptomatic screening programmes), self-examination and symptomatic concerns. Despite mammography-guided fine needle biopsies and morphological/histological studies of core biopsies being the current gold standards for establishing cancerous alterations, these have limitations, not limited to human error and professional experience that can influence diagnosis^3^. Many studies have pointed to the limitations of mammography, offering sensitivity and specificity of 70% to 90%^4^, usually followed up by biopsy to determine whether the abnormality is cancerous^5^.

Metastastic initiation requires invasion of surrounding tissues by malignant cells via a multistep process. Breast cancer usually develops from the epithelial cells lining the terminal duct lobular unit. Disintegration of different junctions functioning to connect epithelial cells to each other allows detachment of epithelial cells and loss of apical-basal polarity. To provide epithelial cells with invading characteristics, a highly conserved cellular program called the ‘epithelial-mesenchymal transition’ (EMT) is induced, restraining epithelial traits in favour of a motile mesenchymal phenotype. The epithelial cells usually interact with the basement membrane via its basal surface to undergo multiple biochemical changes that allow it to take on a mesenchymal cell phenotype, including enhanced migratory capabilities, invasiveness and significantly increased production of extracellular matrix (ECM). As opposed to the EMT viewed as a program that distinctly demarcates cells residing in either an absolute epithelial or mesenchymal condition, the EMT is seen as a plastic program whereby cells progress towards a mesenchymal state, potentially reverting to their epithelial roots once they have invaded a secondary site. Consequently, carcinoma cells are able to re-epithelialize at the metastatic location, being vital for metastatic colonization and development of metastatic outgrowths.

Collagens are major proteins that exist in the extracellular matrix (ECM), the degeneration and penetration of the ECM seeming to be significant processes relating to the morbidity and mortality of cancer^6^. Specifically, association of invasive carcinoma cells with the ECM has been associated with an increase in proteins which then disrupt the ECM, permitting the invasion of carcinoma cells, proliferating steadily to surrounding tissue. Several studies have demonstrated disturbed collagen structures, in particular the degree of deterioration corresponding with invasive carcinoma development in breast^7–9^. Fibrillar collagen types I and III are dominant in ECM^10^, molecular alterations in these being potentially linked with invasive carcinoma. In electron micrograph studies by others, a particular focus has concerned fibrillar collagen in tumour-bearing tissues, degradation in this being observed. The suggestion has been that this could arise from a failure of the altered collagen to aggregate in such a way that would allow formation of the bundles typical of healthy tissue^11,12^. Further to this has been the suggestion that impaired supramolecular organization occurring during cancer progression could provide for pathways engendering invasion of neoplastic cells^13,14^. The organization of the collagen molecules within the tissue can be analyzed through diffraction peaks that reflect the regular packing of the collagen. As one of the prime components of breast tissue, collagen has been studied through a plethora of techniques inclusive of small angle x-ray scattering (SAXS)^5,15,16^. SAXS patterns have previously been analyzed in investigation of the condition of collagen fibrils in breast tissue in attempts to find distinguishable characteristics relatable to breast disease, all containing collagen structures affected by invasive cancer^3,15,17^. Table 1 details the parameters used in previous studies, analytical results demonstrating impact on structural components of breast tissue, particularly in collagen. Table 2 summarizes SAXS breast cancer tissue work of others.

**Table 1:** SAXS features and their relation to the structural components in breast tissue.

SAXS feature	Physically represents
Collagen axial d- spacing	Spacing between the gap-overlap section of the collagen molecules
Collagen axial peak area	Axial peak area defines the combination of peak width and peak amplitude, used herein as an indicator of long-range order in the collagen fibrils. The X-ray constructive interference scattering depends on the abundance of material within the illuminated volume i.e. on parameters such as sample thickness and composition. In general, the larger the area, the greater the degree of long-range order of the samples (i.e. non-lesional).
Collagen axial peak width (full width half maximum- FWHM)	Wide peak corresponds to large variability in the d-spacing. Narrow peaks correspond to low variability in the d-spacing suggesting highly ordered fibrillar collagen overlap spacings
Amorphous scatter	Proportional to the total area per unit mass. Provides information on variation in the electron density of the entire heterogenous sample.
Amorphous scatter between the third and fifth order peak	Integrated intensity between q = 0.2–0.6 nm^−1^. Used to describe the specific surface area of the scatterers.

**Table 2:** Features studied in previous SAXS work.

Year	Research Group	Features observed	Conclusions
2000	Lewis *et al*.^5^	Intensity in axial and Bessel peaks	Scattered intensity in the axial and Bessel peaks were much less in tumour tissue than in normal tissue and benign lesions.
		Spacing of 3rd axial peaks: d-spacing	Larger for benign lesions compared to malignant and normal tissue.
2004	Fernandez *et al*.^25^	Axial period of collagen structure	Axial period of collagen is slightly larger in the fibrils surrounding invasive carcinoma than in healthy areas.
		Average intensity of scattering	The average intensity of scattering from cancerous regions is greater than the intensity from healthy regions.
2005	Round *et al*.^32^	3rd order axial peaks	Differences were observed in normal samples up to 6 cm away from tumour site.
		Equatorial peak area	Obtained difference between normal and benign tissues but no differences observed in tissues invaded by cancer.
2009	Conceicao *et al*.^42^	Diffuse scattering	The diffuse scattering from tumour samples is greater than from benign and normal samples.
		Third- order d- spacing	The third-order axial spacing is greater in malignant lesions than in normal tissue or benign lesions.

It needs to be stressed that the EMT process relates to a relatively brief period of existence, occurring at the onset of malignancy. In the absence of soft tissue mass and associated density changes, detecting early stage changes via mammographic imaging is a particular challenge, evidence of spiculations, calcifcations and disturbed breast architecture being diagnostically equivocal. Further to be acknowledged are the several primary challenges in histopathological evaluation of EMT in human cancer tissue. Difficulties arise in real time tracking of individual cancer cells that have transitioned or are in transition. One issue concerns the staging of EMT, whether it has occurred, whether it has yet to undergo transition or whether the reverse process has occurred, viz the mesenchymal-to-epithelial transition (MET)^18–21^ Nevertheless, the prototypical spindle-shaped morphology of the fibroblasts can be used to advantageous to differentiate between epithelial cancer cells and fibroblasts. Thus said, epithelial cells that have undergone transition are poorly distinguishable from fibroblasts.

The overarching aim of present research on SAXS pattern analysis is to discriminate between different types of breast tissue, healthy, benign or malignant, focusing in particular on those that are EMT negative and those that are EMT positive. In this respect, while numbers of studies have investigated use of SAXS for tissue recognition, none to our knowledge have investigated spectroscopic diagnosis (e.g. by structural component analysis) of EMT breast cancer tissues, a matter of interest in present work. Specifically, we have utilized x-ray synchrotron radiation to gather information on the axial spacing and intensity of scattering patterns, reflective of internal collagenous structure, the scattering profiles being indicative of structural detail at the supramolecular level.

## Methodology

### Breast tissue sample preparation

This work examined breast tissue samples acquired by the Department of Pathology, University of Malaya, obtained from surgical excision or mastectomies. The tissues were acquired following fully informed consent from patients who were subject to surgical treatment, with approval for use in research granted by the Medical Research Ethics Committee, University Malaya Medical Centre in accordance with the International Conference on Harmonization – Guidelines for Good Clinical Practice (ICH-GCP) and Declaration of Helsinki. For the first category of investigations, primary analysis was carried out by the collaborating pathologists on all eight of the tissue samples that were acquired from five patients; these were histologically categorized as EMT-1 to EMT-8 (see Table 3). Following this, SAXS measurements were also carried out on 26 paraffin-wax embedded (subsequently dewaxed) breast tissues from 17 patients (indicated as EMT-9 to EMT-35, as shown in Table 3), the pathological state of these not being revealed to the SAXS investigators (deemed blinded samples). For all cases, the samples were dissected out, obtaining nominal dimensions 5 mm (length) × 5 mm (breadth) × 1 mm (thickness), with two sub-samples taken of each selected case, fixed in formalin solution and kept in Eppendorf tubes (at 10% formaldehyde in water). Prior to SAXS measurements, excess formalin was withdrawn from the tissues and the tissues taped onto a metal frame (10 × 10 mm) on both sides using Kapton tape, a process performed at the synchrotron site.

**Table 3:** The breast cancer tissue samples classified on the basis of pathology.

Research Number	Note	Sample Pathology	Material source
EMT-1	EMT-1 & EMT-2 are tissues from the same patient	EMT positive cancer	FFPE (dewaxed)
EMT-2		Non-lesional	
EMT-3		Fibroadenoma (Benign Tumour)	Formalin-fixed
EMT-4		Fibroadenoma (Benign Tumour)	
EMT-5	EMT- 5 & EMT-6 are tissues from the same patient	Non-lesional	
EMT-6		EMT negative cancer	
EMT-7	EMT- 7 & EMT-8 are tissues from the same patient	Non-lesional	
EMT-8		EMT negative cancer	
EMT-9	EMT-9 & EMT-10 are tissues from the same patient	Blinded (Pathological condition not revealed to SAXS investigator)	FFPE (dewaxed)
EMT-10			
EMT-12			
EMT-13	EMT-13 & EMT-14 are tissues from the same patient		
EMT-14			
EMT-15	EMT-15 & EMT-16 are tissues from the same patient		
EMT-16			
EMT-17			
EMT-18			
EMT-19			
EMT-20			
EMT-21			
EMT-22			
EMT-23	EMT-23 & EMT-24 are tissues from the same patient		
EMT-24			
EMT-25	EMT-25 & EMT-26 are tissues from the same patient		
EMT-26			
EMT-27	EMT-27 & EMT-28 are tissues from the same patient		
EMT-28			
EMT-29	EMT-29 & EMT-30 are tissues from the same patient		
EMT-30			
EMT-31	EMT-31 & EMT-32 are tissues from the same patient		
EMT-32			
EMT-33	EMT-33 & EMT-34 are tissues from the same patient		
EMT-34			
EMT-35			

#### Preparation of breast cancer tissue into FFPE blocks

This study utilized routine patient samples submitted for histopathological examination at the University of Malaya Medical Centre (UMMC), processed as follows. Fresh mastectomy or tumour excision specimens received by the histopathology laboratory following breast cancer surgery were sliced to some 1 cm thickness and immersed in 10% neutral buffered formalin for tissue preservation. The volume of formalin to tissue was about 10:1. Fixation was carried out over a minimum duration of 6 hours, larger specimens (mastectomies included) being commonly fixed overnight. Subsequent to fixation, the specimens were sampled for tumour, surgical margins and any other areas of interest. Each sample was placed into a labelled plastic cassette and kept in 10% neutral buffered formalin to await their processing into formalin-fixed paraffin-embedded (FFPE) blocks.

The completely automated sample processing, from formalin-fixation to paraffin wax (impregnation), was availed using a Leica TP 1020 tissue processor. The sequence was dehydration by alcohol followed by the use of xylene for clearing of alcohol and subsequently replacement of xylene by paraffin wax. In detail, the processing schedule was:

i) Two changes of 10% formalin for 81 minutes each.
ii) Two changes of 95% alcohol for 81 minutes each.
iii) Three changes of 100% alcohol for 81 minutes each.
iv) Two changes of xylene for 81 minutes each.
v) Three changes of wax for 81 minutes each.

After impregnation with paraffin, the tissue was embedded in a desired orientation in the cassette, with a metal mould as the backing upon a hot plate. The cassette was then filled with liquid paraffin and placed on a cold plate to solidify the paraffin. The paraffin block was then popped out of the mould, in so-doing creating a formalin fixed paraffin embedded (FFPE) block in a plastic cassette, which was then ready for microtome sectioning.

#### Dewaxing of the FFPE blocks

The cases recruited for this study were histologically-confirmed breast carcinoma and two cases of benign tumour (fibroadenoma). The EMT status of the breast carcinomas have been determined by the collaborating pathologists based on y scoring of E-cadherin (epithelial) and vimentin (mesenchymal) expression by immunohistochemistry as described in section 2.1.4. For the breast carcinoma cases, selection of study material included additional samples of normal (healthy, non-lesional) tissues from cancer free regions of mastectomies. Exclusion was made of cases with scanty tumour material in the FFPE blocks, the concern arising that future pathology review could be compromised. Dewaxing of the FFPE material for SAXS was performed manually using the following steps:

i) The chosen FFPE blocks were melted down at 65 to 70 °C for about 3 hours.
ii) The sample was retrieved from the melt and dewaxed with xylene for 3–4 hours.
iii) The dewaxed sample was subjected to 100% alcohol cleansing for 30 to 60 minutes.
iv) The sample was then washed under running water until clean.

Dewaxed and rehydrated samples were then subsampled for SAXS. Two subsamples were taken of each selected case, comprising slices of 1 mm thickness. The sliced tissues were then kept in 10% neutral buffered formalin within Eppendorf tubes to await their SAXS examination.

#### Formalin-fixed non-paraffinized tissue

Formalin-fixed non-paraffinized tissue were re-sampled from residual tumour, from mastectomy or tumour excisions of four recently diagnosed cases (2 breast carcinoma and 2 fibroadenoma). These were material that were left over after histopathology diagnosis had been completed and identified for disposal. Duplicate samples from each selected case were of 1 mm thickness. The sliced tissues were then kept in 10% neutral buffered formalin within Eppendorf tubes to await SAXS examination.

#### Determination of EMT status by immunohistochemistry (IHC)

For each study cancer case, microtomed sections from the selected paraffin blocks were stained for immunohistochemical expression of e-cadherin (1:50; Dako: Clone NCH-38) and vimentin (1:500 Dako:Clone V9) using a Ventana BenchMark automated system. Cytoplasmic membrane expressions were semiquantitated for (i) percentage of malignant cells that expressed e-cadherin or Vimentin, as 0 (0–<1%), 1 (1–10%), 2 (11–50%), 3 (more than 50%) and (ii) intensity of staining: 0 (negative), 1(weak), 2 (moderate) and 3(strong). Separately, for both e-cadherin and vimentin, the percentage and intensity scores were then multiplied to obtain a final score. Final scores of 0–4 were classified as low expression and scores of 6–9 classified as high expression. EMT was considered to be present if the cancer showed a low e-cadherin final score, or a high vimentin final score.

#### Determination of tumour size, histological grade, estrogen receptor (ER), progesterone receptor (PR) and HER2 status

It is the routine practice in UMMC for the maximum dimensions (cm) of all excised breast cancers to be recorded in the histopathology reports. The H&E slides of cancers for this study were retrieved and re-evaluated for histological grade based on the modified Bloom and Richardson system which incorporates an evaluation of nuclear pleomorphism, tubule formation and mitotic activity^22^. The presence of calcifications was also noted.

All cancers for this study had been routinely stained for estrogen receptor (ER), progesterone receptor (PR) and HER2 status by IHC using the Ventana BenchMark automated system (see Table 4). The IHC slides were retrieved and expressions for ER, PR and HER2 evaluated according to guidelines of the American Society of Clinical Oncology/College of American Pathologists (ASCO/CAP)^23,24^. Cancers which were ER and PR positive irregardless of HER2 expression were categorized as hormone receptor (HR) positive. Cancers which were negative for ER and PR, and were HER2 positive (i.e. HER2 IHC score 3 or IHC score 2 but confirmed amplified by *in-situ* hybridization) were categorized as HER2 enriched. Cancers which were negative for ER, PR and not HER2 enriched were categorized as triple negative (TN).

**Table 4:** Pathological characteristics of the study samples.

Research number	Pathology	EMT status	Vimentin score	eCadherin score	Size (cm)	Histological grade	Calcifications (Yes/No)	Molecular class (based on ER/PR/HER2 status)	Material for SAXS
EMT-1	Breast cancer	EMT positive	6	3	3.8	3	No	HER2	FFPE (dewaxed) × 2
EMT-2	Normal breast of EMT-1						No		FFPE (dewaxed) × 2
EMT-3	Fibroadenoma (benign tumour)						No		Formalin fixed × 2
EMT-4	Fibroadenoma (benign tumour)						No		Formalin fixed × 2
EMT-5	Normal breast of EMT-6						No		Formalin fixed × 2
EMT-6	Breast cancer	EMT negative	0	6	3.4	3	No	HR	Formalin fixed × 2
EMT-7	Normal breast of EMT-8						No		Formalin fixed × 2
EMT-8	Breast cancer	EMT negative	0	6	3	3	No	HR	Formalin fixed × 2
EMT-9	Breast cancer	EMT positive	9	2	6	3	No	TN	FFPE (dewaxed) × 2
EMT-10	Normal breast of EMT-9						No		FFPE (dewaxed) × 2
EMT-11	Breast cancer	EMT positive	4	3	5.5	2	No	HR	FFPE (dewaxed) × 2
EMT-12	Normal breast of EMT-11						No		FFPE (dewaxed) × 2
EMT-13	Breast cancer	EMT positive	6	3	7	3	No	TN	FFPE (dewaxed) × 2
EMT-14	Normal breast of EMT-13						No		FFPE (dewaxed) × 2
EMT-15	Breast cancer	EMT positive	6	3	8.3	3	No	TN	FFPE (dewaxed) × 2
EMT-16	Normal breast of EMT-15						No		FFPE (dewaxed) × 2
EMT-17	Breast cancer	EMT positive	9	9	2.5	3	No	TN	FFPE (dewaxed) × 2
EMT-18	Breast cancer	EMT negative	0	6	1.5	2	Yes	TN	FFPE (dewaxed) × 2
EMT-19	Breast cancer	EMT positive	9	3	3.5	3	No	TN	FFPE (dewaxed) × 2
EMT-20	Breast cancer	EMT positive	9	9	3.5	3	No	TN	FFPE (dewaxed) × 2
EMT-21	Breast cancer	EMT positive	6	6	3.5	3	No	TN	FFPE (dewaxed) × 2
EMT-22	Breast cancer	EMT positive	0	3	5	3	Yes	HR	FFPE (dewaxed) × 2
EMT-23	Breast cancer	EMT negative	0	9	2	3	No	HR	FFPE (dewaxed) × 2
EMT-24	Normal breast of EMT-23						No		FFPE (dewaxed) × 2
EMT-25	Breast cancer	EMT negative	0	9	4	2	No	HR	FFPE (dewaxed) × 2
EMT-26	Normal breast of EMT-25						No		FFPE (dewaxed) × 2
EMT-27	Breast cancer	EMT negative	0	9	2.7	3	No	HR	FFPE (dewaxed) × 2
EMT-28	Normal breast of EMT-27						No		FFPE (dewaxed) × 2
EMT-29	Breast cancer	EMT negative	0	9	4	3	Yes	HER2	FFPE (dewaxed) × 2
EMT-30	Normal breast of EMT-30						No		FFPE (dewaxed) × 2
EMT-31	Breast cancer	EMT negative	3	9	1.5	1	Yes	HR	FFPE (dewaxed) × 2
EMT-32	Normal breast of EMT-32						No		FFPE (dewaxed) × 2
EMT-33	Breast cancer	EMT negative	0	6	2.5	3	Yes	HER2	FFPE (dewaxed) × 2
EMT-34	Normal breast of EMT-33						No		FFPE (dewaxed) × 2
EMT-35	Breast cancer	EMT negative	3	6	5	3	N	HER2	FFPE (dewaxed) × 2

Key:

EMT status: EMT positive = vimentin scores 6 to 9 and/or ecadherin score 0 to 4; EMT negative = vimentin score 0 to 4 and/or ecadherin score 6 to 9.

Histological grade is according to modified Bloom and Richardson system.

Molecular class: HR = Positive for ER and PR irregardless of HER2 expression; HER2 = HER2 enriched (IHC score 3 or confirmed amplifed by ISH) and negative for ER and PR; TN = Triple negative i.e. negative for ER, PR and not HER2 enriched.

### Small angle X-ray scattering (SAXS) measurement

**Figure 1:**
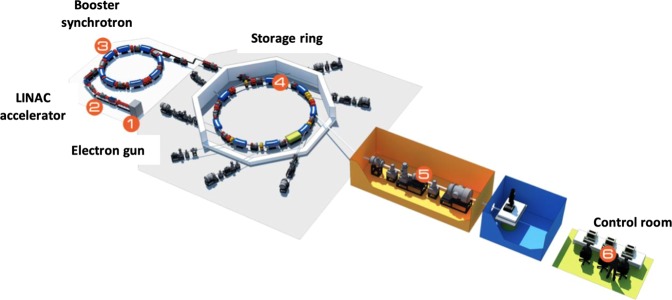
Set-up of the Beamline 1.3 W: SAXS at Synchrotron Light Research Institute (SLRI) in Thailand.

The experiment was conducted at the BL1.3 W: SAXS beamline at the Synchrotron Light Research Institute (SLRI) in Thailand. The experimental setup is as in Fig. 1. An undulator radiation source was used, monochromatized using a Double Multilayer Monochromator (W/B4C), focused with a toroidal mirror to provide an X-ray beam of wavelength 1.379Å (Energy = 9 keV) and a beam size of sample of 2 × 1 mm. The sample-to-detector distance was fixed at 1837.14 mm, a 2-D detector (Rayonix SX165 CCD) providing 2048 × 2048 pixels in recording the scattering pattern. The detector resides inside a vacuum chamber in order to reduce air scattering and absorption losses. The sample-detector-distance allowed recording of momentum transfers within the range 0.06 nm^−1^ ≤ q ≤ 2.50 nm^−1^. To avoid detector saturation a beam stop of 4.5 mm diameter was centrally located adjacent to the detector, radically reducing primary (direct) beam intensity. To calibrate and normalize the SAXS patterns, silver behenate was used as a standard, obtaining the reciprocal space scale of each image^25^. Measurement time was fixed at 5 min, obtaining sufficient scattered photon count while avoiding detector saturation. Once imaged, the tissue samples were returned to the receptacles for continued preservation.

### Data processing

**Figure 2:**
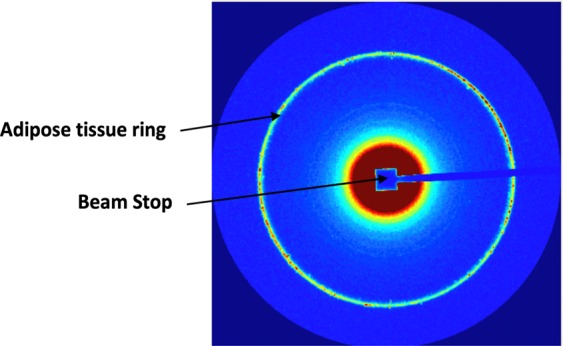
Example of a two-dimensional SAXS diffraction pattern as obtained in the present work.

**Figure 3:**
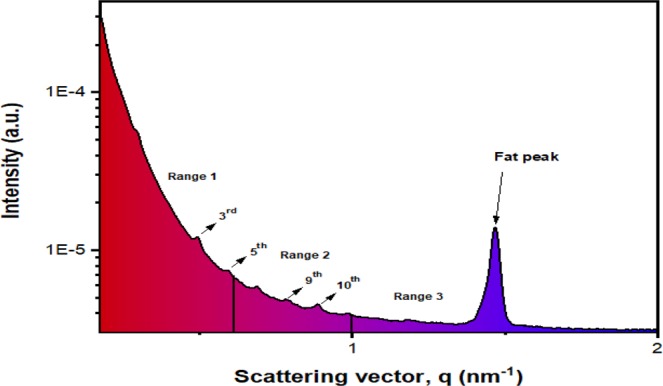
Typical one-dimensional profile extracted from the SAXS signal of non-lesional breast tissue, as obtained in present work. A fat (adipose tissue) peak is found in the momentum transfer range 1.0–2.0 nm^−1^ (labeled ‘Range 3’). The 3^rd^ and 5^th^ order peaks are found in the momentum transfer range 0.2–0.6 nm^−1^ (labeled ‘Range 1’).

Each SAXS image consists of a series of arcs, each arc with a radius commensurate with the momentum transfer q, as can be seen in Fig. 2. Through radial integration, as typically performed for SAXS analysis, supported by SAXSIT software, for each sample the one-dimensional distribution of scattered intensity as a function of momentum transfer was extracted from the two-dimensional SAXS pattern. Of paramount importance in obtaining the scattering profile of the sample itself is that corrections are made to account for sample attenuation and background; Fig. 3 shows an example corrected scattering profile normalized to the incident intensity. Background subtraction is obtained from Eq. 1 and fitting was performed on background subtracted scattering profiles.

1 $${I}_{subbg}={\tilde{I}}_{sam}-(1-c){\tilde{I}}_{bg}$$

1.1 $${\tilde{I}}_{sam}=\frac{{\bar{I}}_{sam}-{\bar{I}}_{dark}}{{T}_{sam}\cdot {i}_{sam,IC}},$$

1.2 $${\tilde{I}}_{bg}=\frac{{\bar{I}}_{bg}-{\bar{I}}_{dark}}{{T}_{bg}\cdot {i}_{bg,IC}}$$

where ${\bar{I}}_{sam}$ is the sample pattern divided by the sample exposure time, $\,{t}_{sam}$, ${\bar{I}}_{bg}$ is the background pattern divided by exposure time, ${{t}}_{bg}$, ${\bar{I}}_{dark}$ is the dark current pattern divided by accumulation time, ${t}_{dark}$. The X-ray intensities which is measured by the ion chamber integrated over the period of the measurements of the sample and background are classified as ${i}_{sam,IC}$, and ${i}_{bg.IC}$, respectively. The sample used herein is solid and therefore the concentration, c was set to zero. Meanwhile, ${T}_{sam}$ is the sample transmission and ${T}_{bg}$ is the background transmission. It should be noted that ${\bar{I}}_{sam}$ is normalized by the exposure time ${t}_{sam}$, sample transmission ${T}_{sam}$ and X-ray intensity measured by ion chamber integrated over the exposure time ${i}_{sam,IC}$.

**Figure 4:**
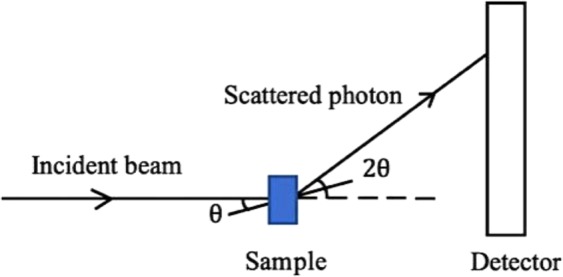
Small angle x-ray scattering geometry. Note: figure drawn in Microsoft powerpoint.

**Figure 5:**
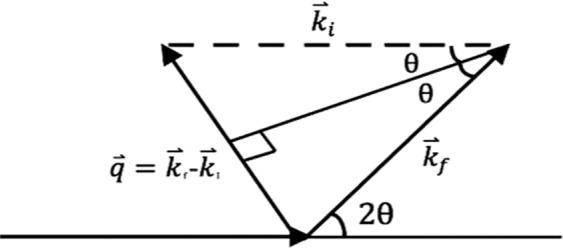
Construction of the scattering vector, $\mathop{{\boldsymbol{q}}}\limits^{{\boldsymbol{\rightharpoonup }}}\,$with incoming photon, ${{\boldsymbol{k}}}_{{\boldsymbol{i}}}$ and the scattered photon, ${{\boldsymbol{k}}}_{{\boldsymbol{f}}}$. Note: figure drawn in Microsoft powerpoint.

SAXS patterns accord with Bragg’s law (Eq. (2)), coherent scattering giving rise to interference and spread as a result of structural periodicity, providing an ability to classify tissues and to identify structures as small as 0.1 nm^3,25^, also being commonly used to characterize macromolecules in low resolution:

2 $$n\lambda =2d\,sin\theta $$

d representing the separation between the periodic scatterers, *θ* being the Bragg angle, equal to half the total scattering angle of the incident monochromatic x-rays, 𝜆 its wavelength and n the order of the scattering maxima. In this way, SAXS studies have provided convincing contrast between healthy and diseased tissues^16,25,26^. In particular, oriented collagen fibrils have distinct SAXS features in the meridional direction due to the axial periodicity^26^, the d-spacing being easily obtained from the axial Bragg peaks. Diffraction pattern differences arise from inhomogeneities in the distribution of electrons in the material. The radial distribution of the observed scattering pattern can also be used to infer particle density and surface area of the sample constituents. Figure 4 illustrates the SAXS geometry; the elastic scattering of the incident photons are conserved and interference of the scattered photons can be sensed by the detector. Scattering data can be presented by relating the observed intensity to the modulus of the scattering vector $\mathop{q}\limits^{\rightharpoonup }$ (see Fig. 5) as described in Eq. (3):

3 $$\mathop{q}\limits^{\rightharpoonup }=\mathop{{k}_{f}}\limits^{\rightharpoonup }-\mathop{{k}_{i}}\limits^{\rightharpoonup }$$

3.1 $$\mathop{|{k}_{i}|}\limits^{\rightharpoonup }=\frac{2\pi }{{\rm{\lambda }}}=\mathop{|{k}_{i}|}\limits^{\rightharpoonup }$$

where the incoming photon, $\mathop{{k}_{i}}\limits^{\rightharpoonup }$ and the scattered photon $\mathop{{k}_{f}}\limits^{\rightharpoonup }$ define $\mathop{q}\limits^{\rightharpoonup }$. From the geometry of Fig. 4, the scattering vector can be described as in Eq. 3.2.

3.2 $$q=\frac{4\pi sin\theta }{{\rm{\lambda }}}$$

Using Eqs. (2) and (3.2), the d-spacing and scattering vector q are related by Eq. (4).

4 $$d=\frac{2\pi n}{q}$$

In this work, the structural components at scattering vector, q = 0.2 nm^−1^ to 1.5 nm^−1^ were investigated as this region corresponds to the region that provide the d-spacing for the collagen. From Fig. 5, it can be deduced that the 3^rd^ and 5^th^ order axial peak were the most intense for non-lesional tissue, benign lesion and EMT negative malignant tissue whereas the 9^th^ and 10^th^ order are the most intense for EMT positive malignant tissue. In regard to the peaks, the software OriginPro2019^27^ was used to fit Gaussian curves to each significant peak of the scattering profiles. From the Gaussian peaks, parameters such as the centre position were determined for each peak. The peak centre corresponds to the x-coordinate of the peak centre, namely the scattering vector, q. The value of the Gaussian center is related to the collagen axial d-spacing, calculated through Bragg’s Law using Eq. 4.

The key advantages of the SAXS method, other than that its generally non-destructive nature, are the availability of extensive variety of sample solution conditions, including the ability to maintain coverage of a wide range of molecular sizes. In particular, it allows one to analyze the structure of native particles in near physiological state, also to examine structural changes in response to an imbalance in external conditions^28^.

### Statistical analysis

From the corrected sample scattering profiles obtained from SAXSIT, parameters associated with structural information have been generated, fittings resulting from use of the software OriginPro2019 (v9.6)^27^. Fitting of the peaks to Gaussians have allowed generation of the parameters: FWHM, amplitudes, centre position and area of the individual peaks. Axial periodicities (d-spacings), extracted from Gaussian peak centres, were obtained using Eq. (4). To determine those variables differentiating between groups, discriminant analysis was performed, R software (Version 3.6.1) being used to perform the Kruskal-Wallis test, with subsequent performance of Dunn’s post hoc test to identify the pairs of tissues which differed significantly for all measurements considered, p values substantiating parameter extractions of practical consequence in constructing a diagnostic model.

## Results and Discussion

### SAXS Analysis of 16 classified breast tissues

**Figure 6:**
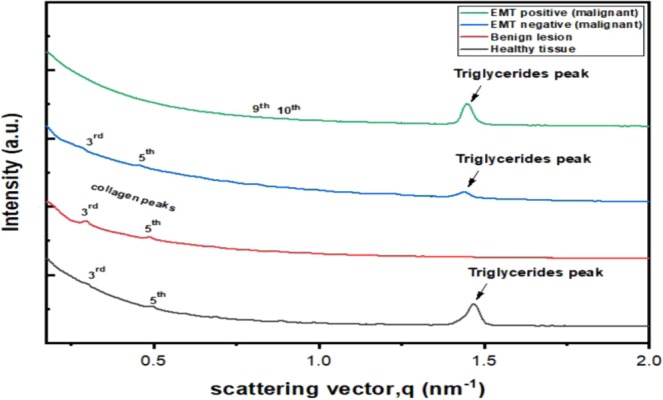
Typical scattering profiles of four groups of breast tissue analyzed in present work.

Figure 6 depicts the scattering profile of each of the tissue groups analyzed in this work, including non-lesional, benign and malignant, EMT negative and EMT positive. Based on the scattering profiles, a number of features were analyzed, as follows: (i) the 3^rd^, 5^th^, 9^th^ and 10^th^ order Bragg peaks located between 0.175–0.200 nm^−1^, corresponding to the axial organization of the collagen fibrils; (ii) the peak at 1.44 nm^−1^ for EMT positive and EMT negative lesions; (iii) 1.46 nm^−1^ for non-lesional tissue, corresponding to the scattering of triglycerides acknowledging this to be one of the most significant of the constituents of lipids. Fat peaks are not present in any of the benign tissue, stroma not containing triglycerides. Finally is a fourth category, a diffuse scattering component manifests a rapidly decreasing, resulting from disordered collagen fibril structures and other constituents of the extracellular matrix.

Observed is that the scattering intensity is greatest in EMT positive malignant tissue, followed by EMT negative malignant tissue and subsequently benign tissue, moderate in intensity, the lowest intensity being for non-lesional tissue. This arises from the propagation of cancer and invasion of normal tissues, leading to changes in collagen fibril structural organization, then causing the endogenous collagen to degenerate and new collagen of diminished structural organization to emerge^8^. Notably, EMT positive lesions manifest at the 9^th^ and 10^th^ order while other tissues manifest in 3^rd^ and 5^th^ order peaks, resolved to be due to the loss of E-cadherin expression in tissues suffering invasion and metastasis^29^. E-cadherin functions to yield mechanically strong adhesive links between cells in the tissue and is crucial in maintaining epithelial morphology^30,31^. It is then replaced by mesenchymal specific N-cadherin and integrins specific for a more transitory adhesion, activating the EMT, invasion and metastasis. In addition, matrix metalloproteinases (MMPs) emerge to degrade the basement membrane, leading to the migration of cancer into the surrounding stroma.

**Figure 7:**
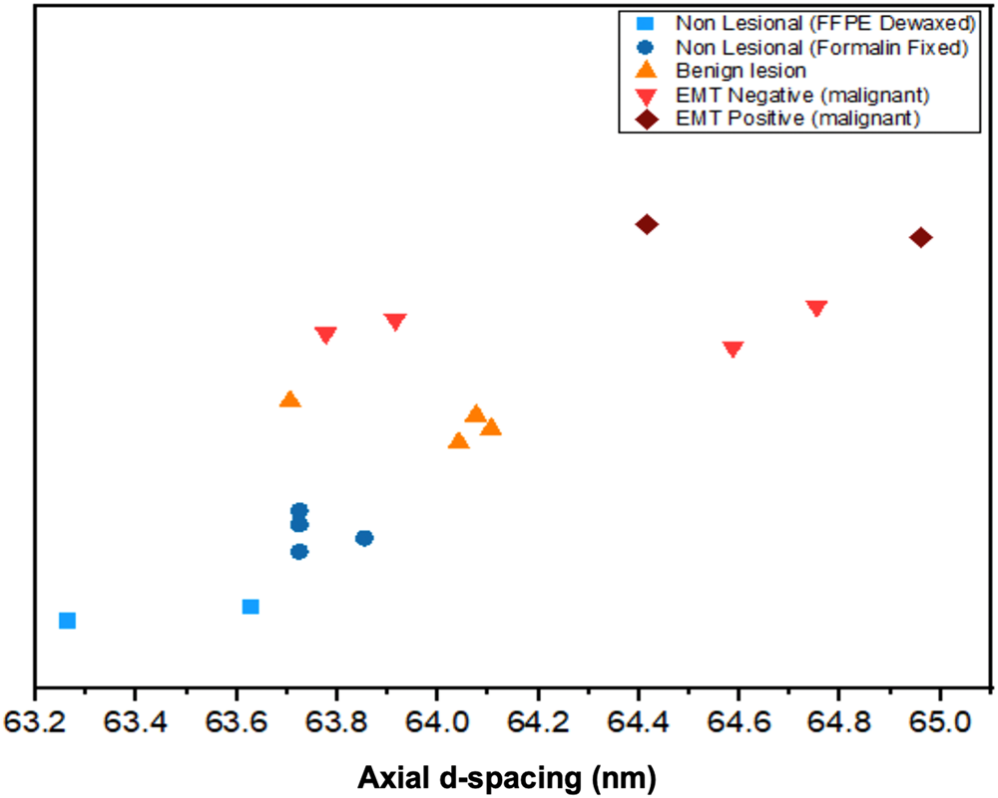
Individual axial d-spacing (nm) points for the 16 groups of classified tissues, including non-lesional, benign, EMT negative and EMT positive. Note: the data points are offset vertically for clarity.

Table 5 shows analysis of the axial spacing, peak area and FWHM (mean and range). Figure 7 demonstrates appreciably larger d-spacing for EMT positive malignant lesion compared to the other tissue types, a consequence of cancer propagation into the breast tissues and increase of N-cadherin expression, the tissue losing cell-cell adhesion as previously mentioned, causing the long-range intermolecular bonding rigidity of collagen to diminish. Further apparent are smaller values of axial peak areas in benign tissue and malignant lesions compared to normal tissue (Table 5 refers). The reduction of the structural order of collagen within lesions and consequent decrease in intensity can be associated with the activity of collagenases, a group of collagen degrading enzymes^32^. Present study has observed benign lesions to be associated with a larger FWHM in the third-order peak than that for negative EMT lesions, also for normal tissue and even positive EMT at the 9^th^ and 10^th^ order (see Table 5), a result similar to that found by previous researchers^16^. Larger FWHM is expected on the grounds that greater variability is to be found in the fibril packing structure of benign lesions.

**Table 5:** Summary peak parameters for 16 classified breast tissues.

Parameters	Group of Tissue	Material Source	Mean ± sd
Axial d-spacing (nm)	Non-lesional (n = 4)	Formalin fixed	63.7 ± 0.1
	Non-lesional (n = 2)	FFPE (dewaxed)	63.4 ± 0.3
	Benign (n = 4)	Formalin fixed	64.0 ± 0.7
	EMT Negative (n = 4)	Formalin fixed	64.3 ± 0.8
	EMT Positive (n = 2)	FFPE (dewaxed)	64.7 ± 0.8
Axial Peak Area (a.u.)	Non-lesional (n = 4)	Formalin fixed	(3.4 ± 1.1) × 10^−8^
	Non-lesional (n = 2)	FFPE (dewaxed)	(4.7 ± 2.2) × 10^−8^
	Benign (n = 4)	Formalin fixed	(2.9 ± 4.4) × 10^−8^
	EMT Negative (n = 4)	Formalin fixed	(4.1 ± 0.9) × 10^−9^
	EMT Positive (n = 2)	FFPE (dewaxed)	(4.7 ± 0.19) × 10^−10^
Third- order FWHM (nm^−1^)	Non-lesional (n = 4)	Formalin fixed	(16.3 ± 0.7) × 10^−3^
	Non-lesional (n = 2)	FFPE (dewaxed)	(15.6 ± 1.0) × 10^−3^
	Benign (n = 4)	Formalin fixed	(18.8 ± 0.1) × 10^−3^
	EMT Negative (n = 4)	Formalin fixed	(13.7 ± 2.9) × 10^−3^
Ninth-order FWHM (nm^−1^)	Non-lesional (n = 4)	FFPE (dewaxed)	(23.6 ± 1.9) × 10^−3^
	Non-lesional (n = 2)	Formalin fixed	(18.9 ± 3.0) × 10^−3^
	Benign (n = 4)	Formalin fixed	(15.0 ± 0.6) × 10^−3^
	EMT Negative (n = 4)	FFPE (dewaxed)	(3.7 ± 1.8) × 10^−3^
	EMT Positive (n = 2)	FFPE (dewaxed)	(4.5 ± 2.7) 10^−3^
Tenth-order FWHM (nm^−1^)	Non-lesional (n = 4)	FFPE (dewaxed)	(24.1 ± 2.4) × 10^−3^
	Non-lesional (n = 2)	Formalin fixed	(14.8 ± 1.5) × 10^−3^
	Benign (n = 4)	Formalin fixed	(12.1 ± 1.4) × 10^−3^
	EMT Negative (n = 4)	FFPE (dewaxed)	(16.0 ± 4.4) × 10^−3^
	EMT Positive (n = 2)	FFPE (dewaxed)	(13.8 ± 5.2)10^−3^
Triglyceride FWHM (nm^−1^)	Non-lesional (n = 4)	Formalin fixed	(53.7 ± 13.6) × 10^−3^
	Non-lesional (n = 2)	FFPE (dewaxed)	(40.0 ± 10.0) × 10^−3^
	Benign (n = 4)	Formalin fixed	No peak
	EMT Negative (n = 4)	Formalin fixed	(88.8 ± 15.4) × 10^−3^
	EMT Positive (n = 2)	FFPE (dewaxed)	(49.1 ± 27.9) × 10^−3^
Triglyceride axial peak area (a.u.)	Non-lesional (n = 4)	Formalin fixed	(0.02 ± 0.01) × 10^−6^
	Non-lesional (n = 2)	FFPE (dewaxed)	(0.4 ± 0.02) × 10^−6^
	Benign (n = 4)	Formalin fixed	No peak
	EMT Negative (n = 4)	Formalin fixed	(0.039 ± 0.006)10^−6^
	EMT Positive (n = 2)	FFPE (dewaxed)	(0.23 ± 0.008)10^−6^

### SAXS Analysis of 51 blinded breast tissues

In regard to differences in axial d-spacing between non-lesional and malignant breast tissues, as seen in the summary results of the 16 classified breast tissues investigation (Fig. 7), this is associated with the disruption of collagen fibrils structure. Note that collagen is a long-range periodic structure, repeated along the fibril axis, containing molecules packed laterally to each other with hydrogen bonds, binding to each other longitudinally in a staggered arrangement. Tissue remodeling is an essential step during the malignant process, transformation of epithelial cells being associated with metalloproteinases in collagen degradation by synthesis of fibrillary and non-fibrillary matrix proteins^33^. Extensive alteration of the ECM has been observed, collagen derangement being attributed to enzymatic degradation and altered neosynthesis. It is evident that at the supramolecular level changes in collagen in tissue invaded by cancer can be described quantitatively from the SAXS patterns, paving the way for tissue classification and improved understanding of mechanisms of cancer growth.

In this section, using a similar experimental SAXS setup to that above, the study has been repeated for 51 blinded breast tissues (FFPE (dewaxed)), seeking to distinguish differences between the tissues groups, non-lesional and malignant (including EMT negative and EMT positive breast tissue). Biological tissue SAXS studies^3,17,25,34^ often place emphasis on odd-ordered Bragg peaks in obtaining the periodicity within the collagen present in the sample. It is apparent that the odd peaks are larger than the neighboring even peaks, revealing a qualitative feature of the structure. The nth order Bragg peak derives from the nth Fourier component of the electron density unit cell^35^, contributing to the odd cosine waves with feature that the amplitude is of the opposite sign at the centre of the unit interval than it is at the edges. This is a known feature of the structure of long-chain hydrocarbons exist in extracellular matrix (ECM).

**Figure 8:**
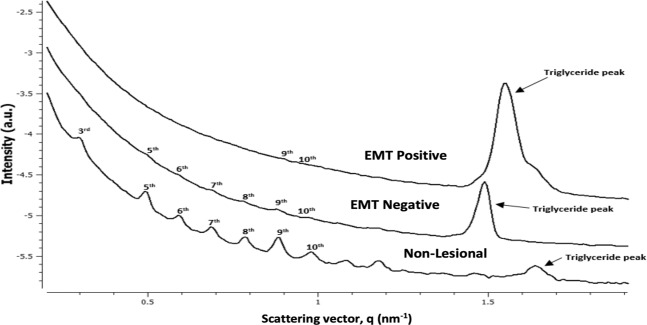
Wide scattering profile of blinded samples, including non-lesional, malignant; EMT negative and EMT positive breast tissues.

Highly-ordered healthy collagen exhibit sharp and prominent peaks from the 3^rd^ to the 10^th^ order; conversely, axial ordering gradually disappears in invaded regions, most particularly for EMT positive tissues, as in Fig. 8, demonstrating greater degradation in diseased tissues than surrounding healthier tissue. Variations in axial periodicity result from a change in crimping angle^36^, also if the normal type I collagen molecule is replaced by the trimer in which the three chains are identical^8^ and high concentration of type III is ruled out^36^. It has been suggested that the contact of epithelial cells with collagen I contributes to increased cell motility accompanying EMT, critical in disease progression^37^. For invaded regions the scattering intensity has been observed to be significantly greater, the triglyceride peak also being broader, EMT positive breast cancer tissue in particular where collagen fibrils can suffer “peeling off”, corresponding to large increase in the specific surface area per unit volume of scatterers, agreeing with the findings of Fernandez *et al*.^25^.

Fibrils orientation was determined from the maximum intensity of the collagen peaks; the calculated axial peak area and FWHM are detailed in Table 6. Small but systematic differences are seen in axial d-spacing, the period of collagen being slightly greater in fibrils surrounding invasive carcinoma cells than in non-lesional areas, interpreted as an increase in axial periodicity with malignancy. It has also been reported^38^ that breast tumours display aberrant collagen bundle organization in the tumour stroma. The FWHM and peak amplitudes show significant differences between diseased and non-lesional breast tissues. In the case of collagen, the FWHM is related to the distribution of axial d-spacings in the fibrillar collagen and the amount of variability in the distribution of particle sizes in the sample. The FWHM of the investigated samples show a general trend of decreasing FWHM with severity of disease, in particular EMT positive. A larger width would depict more variability in the distance between the collagen fibrils compared to a peak with a smaller width. Here, it is important to keep in mind that the peak area is the incorporation of the peak width and peak amplitude. The axial peak area describes the extent of the long-range order in the packing structure of the collagen, arising from the fact that x-ray scattering involves constructive interference, whereby the larger the area, the greater the degree of long-range order of the samples. In Table 6, it can be observed that the axial peak area decreases with malignancy. The reduction of the peak area is possibly caused by the action of collagen-degrading enzymes known as collagenases as found in other work^32^. In the case of a greater degree of ordering (i.e. non-lesional tissue) this will be portrayed as a combination of broader width and greater amplitude, thus of larger axial peak area, vice-versa in the less ordered malignant tissue. It should be noted, as stated in Table 1, that peak area depends on the abundance of material within sample, thickness and composition in particular. The dissected-out samples are of nominal dimensions 5 mm (length) × 5 mm (breadth) × 1 mm (thickness), assumed to be sufficiently equal across samples to allow use of the peak area as parameter for quantitative comparisons.

**Table 6:** SAXS peak parameters for the 51 blinded individual breast tissue cases, each in FFPE (dewaxed) condition. Pathology reporting was performed after conduct of the experiment.

Research Number	Order range	FWHM Order range (nm^−1^)	Axial peak area (a.u.) range	Axial d- spacing (nm) average	FWHM Triglyceride (nm^−1^)	Axial peak area Triglyceride (a.u.)
**Non-Lesional Breast Tissue**						
10A	3,5,9,10	0.020–0.014	5.20 × 10^−7^–4.64 × 10^−9^	63.5	0.05	6.69 × 10^−6^
10B	3,5,9,10	0.021–0.030	6.81 × 10^−7^–3.73 × 10^−8^	62.5	0.08	8.63 × 10^−7^
12A	3,5,9,10	0.015–0.044	4.93 × 10^−8^–1.23 × 10^−8^	63.8	0.06	1.03 × 10^−6^
12B	3,5,9,10	0.018–0.027	1.69 × 10^−7^–7.35 × 10^−8^	63.6	0.04	1.96 × 10^−6^
14A	3,5,9,10	0.012–0.004	1.18 × 10^−9^–4.36 × 10^−11^	62.8	0.03	5.30 × 10^−6^
14B	5,9,10	0.021–0.013	7.69 × 10^−9^–2.10 × 10^−9^	64.7	0.06	6.34 × 10^−7^
16A	5,9,10	0.026–0.017	2.77 × 10^−8^–2.88 × 10^−9^	63.5	0.04	2.68 × 10^−7^
16B	5,9,10	0.022–0.012	5.76 × 10^−9^–7.65 × 10^−10^	64.8	0.04	2.95 × 10^−7^
24A	3,5,9,10	0.021–0.028	4.08 × 10^−7^–1.71 × 10^−8^	63.3	0.05	3.18 × 10^−8^
24B	5,9,10	0.024–0.027	5.61 × 10^−8^–8.86 × 10^−9^	63.7	0.11	8.53 × 10^−7^
26A	5,9,10	0.025–0.006	1.38 × 10^−8^–2.68 × 10^−10^	64.4	0.03	3.74 × 10^−8^
26B	5,9,10	0.019–0.016	3.89 × 10^−8^–2.24 × 10^−9^	63.8	0.05	7.26 × 10^−6^
28A	5,9,10	0.022–0.027	6.82 × 10^−8^–7.97 × 10^−9^	63.7	0.06	7.88 × 10^−7^
28B	5,9,10	0.020–0.026	3.44 × 10^−8^–5.07 × 10^−9^	63.8	0.05	1.78 × 10^−6^
30A	5,9,10	0.023–0.023	2.87 × 10^−8^–5.08 × 10^−9^	64.2	0.06	1.20 × 10^−6^
30B	3,5,9,10	0.020–0.026	2.94 × 10^−7^–1.60 × 10^−8^	63.3	0.06	3.73 × 10^−7^
32A	3,5,9,10	0.021–0.030	2.56 × 10^−7^–9.35 × 10^−9^	63.0	0.04	2.34 × 10^−6^
32B	5,9,10	0.023–0.012	2.46 × 10^−8^–7.69 × 10^−10^	63.4	0.05	1.26 × 10^−6^
34A	3,5,9,10	0.007–0.025	1.10 × 10^−7^–2.54 × 10^−10^	63.6	0.02	1.75 × 10^−9^
34B	5,9,10	0.014–0.029	1.51 × 10^−8^–1.09 × 10^−9^	63.9	0.06	1.08 × 10^−7^
**Emt Negative Breast Cancer Tissue**						
18A	3,5,9,10	0.014–0.006	7.31 × 10^−8^–4.35 × 10^−10^	63.8	0.08	3.63 × 10^−7^
18B	3,5,9,10	0.017–0.015	2.43 × 10^−7^–2.01 × 10^−9^	63.6	0.04	4.99 × 10^−7^
23A	3,5,9	0.014–0.029	1.13 × 10^−7^–4.05 × 10^−8^	63.9	0.04	5.00 × 10^−8^
23B	5,9,10	0.007–0.017	1.64 × 10^−9^–1.78 × 10^−10^	63.4	0.03	1.38 × 10^−8^
25A	5,9,10	0.011–0.015	1.23 × 10^−9^–9.32 × 10^−10^	64.4	0.04	1.15 × 10^−8^
25B	5,9,10	0.009–0.014	1.99 × 10^−9^–4.03 × 10^−10^	63.8	0.05	3.73 × 10^−7^
27A	9,10	0.023–0.014	8.40 × 10^−9^–2.86 × 10^−9^	63.8	0.06	6.51 × 10^−7^
27B	9,10	0.009–0.015	1.16 × 10^−9^–2.58 × 10^−9^	64.5	0.08	2.63 × 10^−7^
29A	5,9,10	0.016–0.029	2.73 × 10^−8^–9.25 × 10^−9^	64.0	0.03	8.16 × 10^−7^
29B	5,9,10	0.023–0.011	1.12 × 10^−7^–2.08 × 10^−9^	63.3	0.04	4.96 × 10^−6^
31A	3,5,9,10	0.020–0.024	3.82 × 10^−7^− 7.40 × 10^−9^	63.2	0.05	5.74 × 10^−7^
31B	No peaks					
33A	9,10	0.010–0.010	1.22 × 10^−9^–1.12 × 10^−9^	64.3	0.04	1.95 × 10^−6^
33B	5,9,10	0.019–0.006	2.78 × 10^−8^–2.51 × 10^−10^	64.3	0.05	1.67 × 10^−7^
35A	5,9	0.020–0.018	6.75 × 10^−8^–1.08 × 10^−8^	63.5	0.04	3.20 × 10^−7^
35B	5,9	0.023–0.018	1.20 × 10^−7^− 9.06 × 10^−9^	64.1	0.06	7.18 × 10^−7^
**Emt Positive Breast Cancer Tissue**						
9A	9,10	0.012- 0.019	2.61 × 10^−9^–2.18 × 10^−9^	65.2	0.08	1.02 × 10^−6^
9B	9,10	0.009–0.005	6.31 × 10^−10^–1.34 × 10^−10^	64.7	0.09	2.45 × 10^−7^
13A	9,10	0.01–0.021	1.31 × 10^−9^–2.01 × 10^−9^	65.9	0.03	1.27 × 10^−6^
13B	5,9	0.012–0.013	8.09 × 10^−9^–2.75 × 10^−9^	64.4	0.04	3.47 × 10^−6^
15A	9,10	0.004–0.005	1.12 × 10^−10^–1.78 × 10^−10^	65.7	0.04	1.64 × 10^−7^
15B	9,10	0.01–0.007	1.79 × 10^−9^–1.74 × 10^−9^	65.0	0.04	3.43 × 10^−6^
17A	5,9,10	0.02–0.013	2.22 × 10^−8^–1.02 × 10^−9^	63.7	0.05	1.75 × 10^−6^
17B	5,9,10	0.019 – 0.017	1.82 × 10^−8^–3.2 × 10^−9^	63.0	0.09	1.83 × 10^−6^
19A	9,10	0.013–0.008	1.71 × 10^−9^–5.67 × 10^−10^	64.7	0.04	4.42 × 10^−8^
19B	9,10	0.012–0.004	2.02 × 10^−10^–6.08 × 10^−10^	64.7	0.06	1.48 × 10^−7^
20A	9,10	0.006–0.006	5.21 × 10^−10^–3.58 × 10^−10^	65.6	0.06	1.74 × 10^−6^
20B	9,10	0.007–0.003	5.31 × 10^−10^–1.41 × 10^−10^	65.5	0.04	1.63 × 10^−6^
21A	5,9	0.018–0.017	7.96 × 10^−9^–4.68 × 10^−9^	63.6	0.07	3.97 × 10^−7^
21B	5,9,10	0.0272–0.004	2.45 × 10^−8^–3.01 × 10^−10^	64.2	0.07	1.17 × 10^−6^
22A	5,9,10	0.016–0.002	8.14 × 10^−9^–1.23 × 10^−10^	63.7	0.09	4.98 × 10^−7^
22B	5,9,10	0.014–0.007	9.10 × 10^−9^–4.08 × 10^−10^	64.5	0.07	2.13 × 10^−6^

While 5^th^, 9^th^ and 10^th^ axial order are present in both EMT negative and EMT positive, an observed effect of degradation in the poorly ordered collagen of EMT positive is reduction of FWHM and axial peak area (Table 6). Comparison can also be made with the earlier work on the classified 16 tissues, the d-spacing being within the same range. Special care needs to be taken to include all key parameters, indicators arising from change in collagen fibril packing (axial peak d-spacing) and the amount of collagen in the tissue (integrated intensities and area), correlating with morphological features and cell types of the tissues. For instance, while EMT-17, EMT-18 and EMT-31 produce typical scattering spectra for non-lesional tissue, the axial area of EMT-17 for all d-spacing orders are much less than non-lesional and EMT negative tissues; additionally, the FWHM of all d-spacing orders for EMT-18 are much less than non-lesional tissue while EMT-31 shows greater d-spacing than non-lesional tissue. Accordingly, EMT-17 is classified as EMT positive while EMT-18 and EMT-31are classified as EMT negative. As with other studies, present studies find collagen fibril d-spacing, axial peak amplitude and the area of the lateral peaks equatorial peak features to be distinguishing parameters.

Table 7 summarises the peak parameters of the several tissue types investigated. As expected the FWHM of triglyceride peak is greater in EMT positive tissues compared to non-lesional tissues. Of interest are triglycerides, fat being a major source of energy for growth and membrane synthesis in solid tumours^39,40^, the proportions of fat and fibrous tissue present in breast being associated with enhanced cell migration and aggressiveness, characteristic of metastatic cells, suggestive of activation of *de novo* lipogenesis in EMT induced cells^41^.

**Table 7:** Summary peak parameters for the 51 blinded breast tissues, each in FFPE (dewaxed) condition.

SAXS Parameter	Group of Tissue	Mean ± sd
Axial d-spacing (nm)	Non-lesional	63.6 ± 1.0
	EMT Negative	62.3 ± 9.9
	EMT Positive	64.5 ± 1.5
Axial Peak Area (a.u.)	Non-lesional	(6.8 ± 12.5) × 10^−8^
	EMT Negative	(4.3 ± 7.7) × 10^−8^
	EMT Positive	(0.5 ± 0.61) × 10^−8^
Third-order FWHM (nm^−1^)	Non-lesional	(19.6 ± 2.3) × 10^−3^
	EMT Negative	(17.2 ± 2.9) × 10^−3^
	EMT Positive	No peak
Fifth-order FWHM (nm^−1^)	Non-lesional	(22.3 ± 3.4) × 10^−3^
	EMT Negative	(18.5 ± 4.9) × 10^−3^
	EMT Positive	(16.7 ± 3.4) × 10^−3^
Ninth- order FWHM (nm^−1^)	Non-lesional	(25.1 ± 8.3) × 10^−3^
	EMT Negative	(18.1 ± 7.4) × 10^−3^
	EMT Positive	(13.6 ± 8.2) × 10^−3^
Tenth- order FWHM (nm^−1^)	Non-lesional	(20.2 ± 10.4) × 10^−3^
	EMT Negative	(13.7 ± 7.0) × 10^−3^
	EMT Positive	(8.7 ± 6.1) × 10^−3^
Triglyceride FWHM (nm^−1^)	Non-lesional	(51 ± 19.4) × 10^−3^
	EMT Negative	(49 ± 15.5) × 10^−3^
	EMT Positive	(60 ± 20.7) × 10^−3^
Triglyceride Axial Peak Area (a.u.)	Non-lesional	(1.7 ± 2.17) × 10^−6^
	EMT Negative	(0.8 ± 1.25) × 10^−6^
	EMT Positive	(1.3 ± 1.08) × 10^−6^

**Figure 9:**
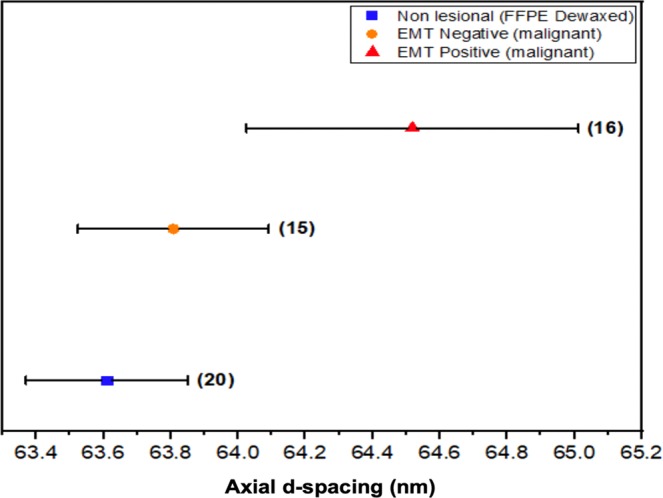
Mean axial d-spacing (nm) for the 51 blinded groups of tissues; non-lesional and malignant; EMT negative and EMT positive. The number of samples are shown in the brackets. Note: error bars represent standard deviation. Note: the data points are offset vertically for clarity.

Lewis *et al*.^5^ acquired SAXS images by averaging over the whole tissue sample while SAXS images acquired by Fernandez *et al*.^25^. resulted from the selecting of several points on the tissue sample (1–0.25 mm intervals), each image considered a discrete observation relative to the sample pathology. The axial d-spacing of collagen in benign and non-lesional breast tissues reported by Fernandez *et al*.^25^. are 65.3 ± 0.2 nm and 65.0 ± 0.1 nm, respectively. On the other hand, the d-spacing of collagen found by Lewis *et al*.^5^ for invasive carcinoma and normal tissues is within the range 64.7 nm–65.2 nm, whereas a smaller d-spacing was obtained for benign tissues between 64.4 and 64.7 nm. The present study found the d-spacing in invasive EMT positive, at 64.5 ± 0.2 nm, to be larger than in histopathologically invasive EMT negative at 63.8 ± 0.1 nm and non-lesional d = 63.6 ± 0.1 nm tissues, as illustrated in Fig. 9 (the error bars in the graph represent the standard deviation). It should be noted that the formalin fixation samples of present investigation are found to change and reduce the absolute d-spacing of the collagen fibril in comparison to previous research, although it does not alter the relative differences between non-lesional and malignant tissues, being of great value since the transport and restoration of samples is greatly facilitated by formalin fixation. The larger standard deviation of the d-value in invaded regions (EMT positive) can be explained by the weak Bragg reflections and higher background.

Kruskal-Wallis test has been performed to examine if the four types of tissues; non-lesional, benign, EMT negative and EMT positive differed significantly by considering d-spacing, axial area, FWHM, FWHM triglyceride and axial area triglyceride (see Table 8). It has been found that differences exist between the four tissues for d-spacing, axial area and FWHM. Dunn’s post hoc test was subsequently performed to identify the pairs of tissues which differed significantly for all five measurements considered (see Table 9). The p-values for axial area triglyceride and FWHM triglyceride showed insignificant differences for all four tissues. The EMT positive – non-lesional pair showed significant differences for d-spacing, axial area and FWHM while EMT negative – EMT positive appeared to be significantly different for axial area.

**Table 8:** Kruskal-Wallis test for differences in non-lesional, benign, EMT positive and EMT negative tissues according to measurements.

Measurements	p-value
d-spacing	0.014 (**)
Axial peak area	0.007 (**)
FWHM	0.002 (**)
Axial area triglyceride	0.049 (#)
FWHM triglyceride	0.065 (##)

Note: (**) highly significant, (*) significant, **(##)** provisional significance.

**Table 9:** Dunn’s post hoc test to assess the significant differences in measurements between non-lesional, benign, EMT negative and EMT positive tissues.

	p-value				FWHM triglyceride
	d-spacing	Axial peak area	FWHM	Axial area triglyceride	
Benign – EMT negative	0.752	0.828	0.483	0.152	0.138
Benign – EMT positive	0.475	0.134	0.345	0.057(##)	0.053(##)
Benign – non-lesional	0.461	0.940	0.530	0.085	0.127
EMT negative – EMT positive	0.144	0.043(*)	0.383	0.198	0.247
EMT negative – non-lesional	0.752	0.600	0.028(**)	0.407	0.724
EMT positive – non-lesional	0.008(**)	0.005(**)	0.001(**)	0.450	0.338

Note: (**) highly significant, (*) significant, **(##)** provisional significance.

**Figure 10:**
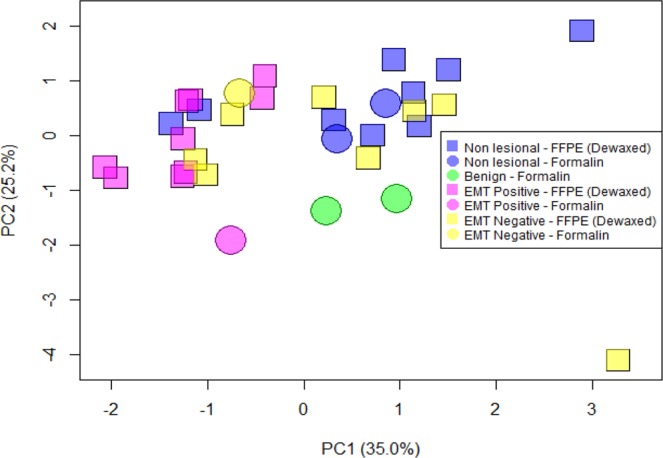
PCA plot of all studied samples; non-lesional, benign, EMT negative and EMT positive tissues.

In present work the healthy and unhealthy tissues from the same patient were considered as independent. The Cook’s distance was calculated and two outliers were detected, belonging to patients 10 and 30 – both with non-lesional tissues in the FFPE (dewaxed) condition. Data from patients 10 and 30 were omitted before further analysis was performed. General trends in the data from measurements on the tissues were studied via principal component analysis (PCA) (See Fig. 10) and the methods used, as described below:

1. Non-lesional - FFPE (dewaxed) (N = 9); Formalin fixed (N = 2)
2. Benign - Formalin fixed (N = 2)
3. EMT negative - FFPE (dewaxed) (N = 8); Formalin fixed (N = 2)
4. EMT positive - FFPE (dewaxed) (N = 9); Formalin fixed (N = 1)

Figure 10 indicates the presence of two clusters along the PC1 axis, one in the direction of negative PC1 (EMT positive) and another in the opposite direction (non-lesional). Two observations with non-lesional tissues belonging to patients 14 and 26 are seen in the EMT positive cluster. The PCA plot explains 60.2% of the total variation where 35.0% of the total variation is described by axis 1 while 25.2% is described by axis 2. An extreme observation belonging to patient 31 (EMT negative) whose D-spaceB measurement failed to show peak readings is seen in the PCA plot. Patient 24 (non-lesional) also appeared to be an outlier due to high readings of axial peak area and FWHM. These two observations were retained in the data as they did not affect the results of the Kruskal-Wallis test and the Dunn post hoc test.

## Conclusion

It is shown that the SAXS technique is able to produce rapid high-quality interpretable diffraction (structural) data from breast excision specimens, employed herein to seek differentiation between normal, benign and malignant breast tissues, the latter consisting of EMT negative and EMT positive cancers. From the Kruskal-Wallis and Dunn post hoc tests, we have found that the difference between the d-spacing of the EMT positive lesion and non-lesional tissue is highly significant (p < 0.008). The respective d-spacings for the various tissues are: for EMT positive lesion, in the range 64.5–64.7 nm; for EMT negative lesions, FFPE (dewaxed) and formalin fixed, 63.8 nm and 64.3 nm, respectively; for benign lesions 64.0 nm; for non-lesional tissues FFPE (dewaxed) 63.7 nm and; for non-lesional tissue (formalin-fixed) in the range 63.4–63.6 nm. For EMT positive breast tissue, the disturbed collagen structure is most notable in the 9^th^ and 10^th^ order. Thus said, present study has found that axial d-spacing on its own does not offer the sufficiency needed in determining early breast disease. Instead, other features, such as, the amplitude of the third, fifth, nine and tenth-order axial Bragg peaks are needed, together with the magnitude of the integrated intensity and the full-width at half-maximum of the fat peak, offering significant differences between tissue types. As such, the presence of disease is thought to be best represented by a combination of factors, rather than any single specific trait. Of particular note in regard to formalin fixation of samples is that no alteration is observed to occur in the relative differences in collagen d-spacing between non-lesional and malignant tissues. This is a matter of great importance given that preserved-sample and also retrospective study of samples is greatly facilitated by formalin fixation. Present findings indicate that molecular structure characteristics of breast tissue, obtained at the SAXS (supramolecular) level, can be used as markers of disease progression, SAXS analysis offering potential as a viable diagnostic procedure in support of breast clinics.
